# Supplementary material for: Machine learning analysis of volatolomic profiles in breath can identify non-invasive biomarkers of liver disease: A pilot study
Source: PLoS One. 2021 Nov 30;16(11):e0260098. doi: 10.1371/journal.pone.0260098 (PMC8631657; doi:10.1371/journal.pone.0260098)

Supplementary Figure 1: Data pre-processing pipeline. A custom pre-processing pipeline was used to derive DF specific chromatograms and determine peak ion intensity values at individual retention times from the raw data output files.

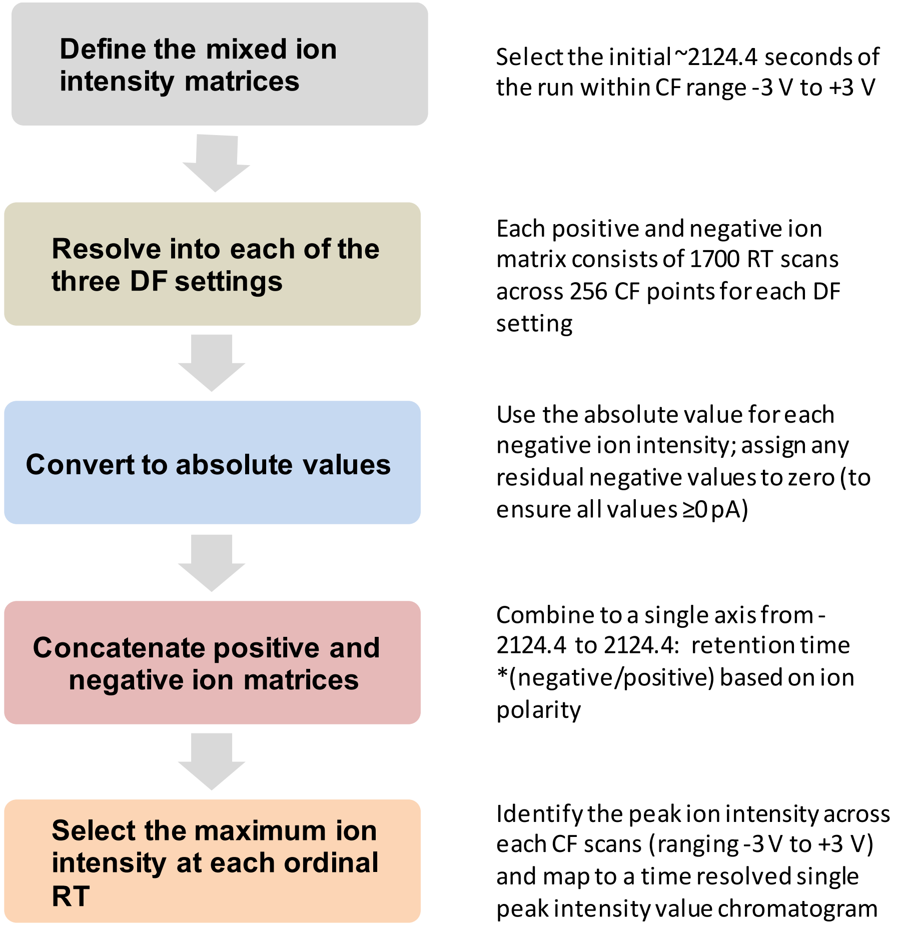

Supplement: S1 Fig — (PDF) [file pone.0260098.s001.pdf]
