# Supplementary material for: Machine learning analysis of volatolomic profiles in breath can identify non-invasive biomarkers of liver disease: A pilot study
Source: PLoS One. 2021 Nov 30;16(11):e0260098. doi: 10.1371/journal.pone.0260098 (PMC8631657; doi:10.1371/journal.pone.0260098)

Supplementary Figure 2: Separation based chromatograms from breath analysis of patients at different disease stages. Time resolved chromatograms were obtained at different DF settings. Stage dependent changes in intensity are apparent at each DF setting. Green lines: stage 0, red lines: stage 1 or 2, and purple lines; stage 3.

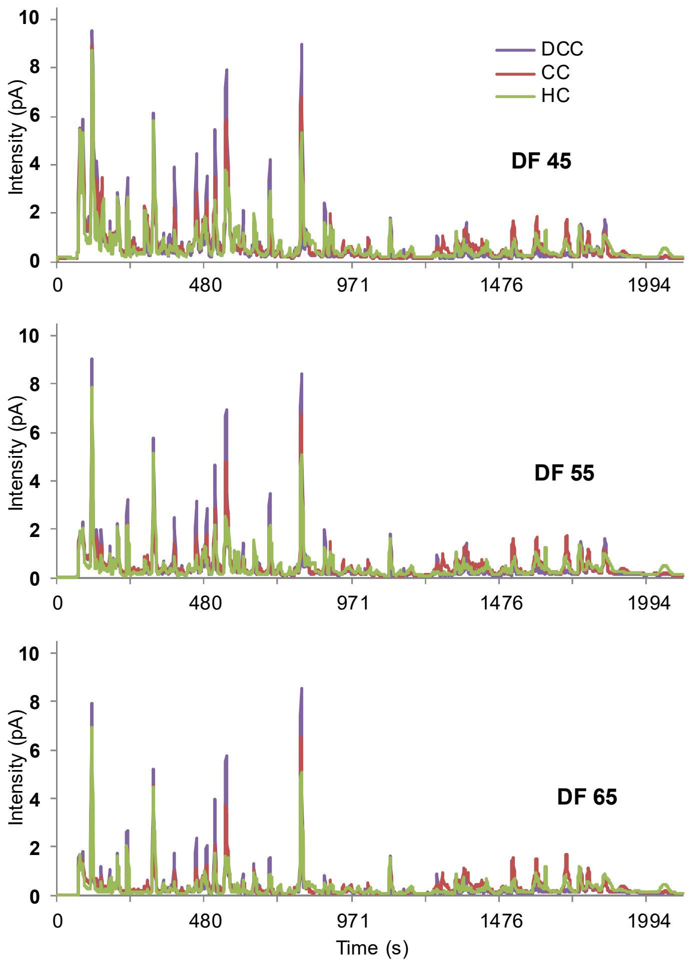

Supplement: S2 Fig — (PDF) [file pone.0260098.s002.pdf]
