# Supplementary material for: Machine learning analysis of volatolomic profiles in breath can identify non-invasive biomarkers of liver disease: A pilot study
Source: PLoS One. 2021 Nov 30;16(11):e0260098. doi: 10.1371/journal.pone.0260098 (PMC8631657; doi:10.1371/journal.pone.0260098)

Supplementary Figure 3: Variability of molecular features with age or etiology. Each plot represents the median standard deviation (SD) of MF peak area for a single MF at DF45 within biological replicates at a given age (A) or etiology (B). The underlying condition represents samples from individuals without liver disease (normal), or with alcoholic liver disease (ALD), chronic hepatitis C virus (HCV), hereditary hemochromatosis (HHC), Non-alcoholic steatohepatitis (NASH) or primary sclerosing cholangitis (PSC).

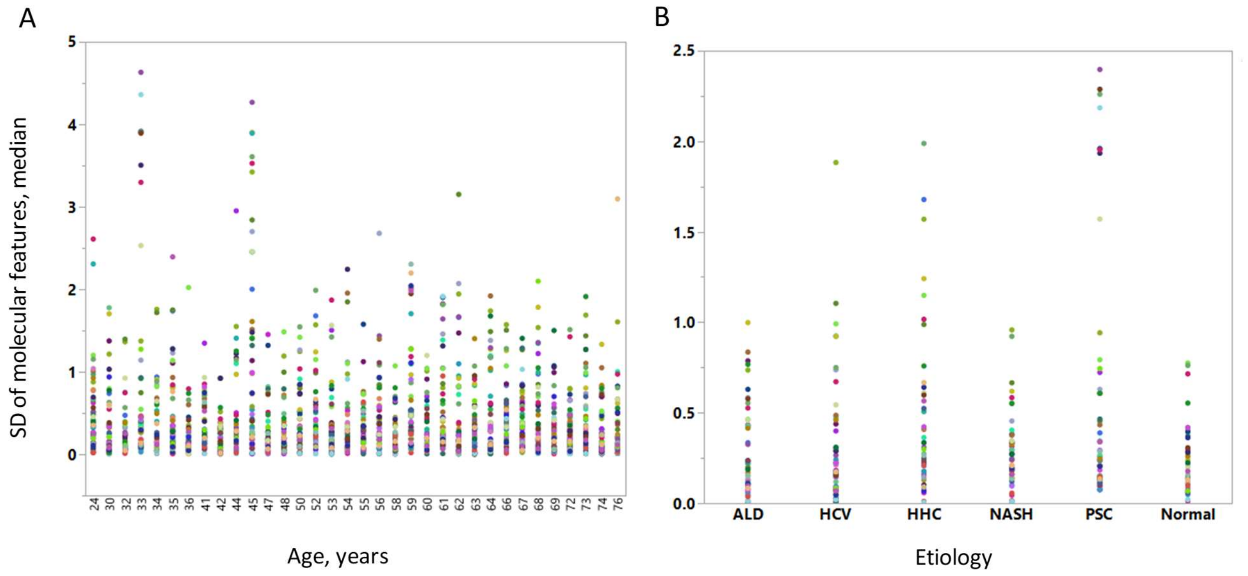

Supplement: S3 Fig — (PDF) [file pone.0260098.s003.pdf]
