# Supplementary material for: Machine learning analysis of volatolomic profiles in breath can identify non-invasive biomarkers of liver disease: A pilot study
Source: PLoS One. 2021 Nov 30;16(11):e0260098. doi: 10.1371/journal.pone.0260098 (PMC8631657; doi:10.1371/journal.pone.0260098)

Supplementary Figure 4: Inter-individual variability in separation chromatograms

Raw breath samples were analyzed an data matrices obtained at DF 45 were run through RESNET-50, a pre-trained image recognition CNN, to examine inter-individual variability by comparing intermediate prediction values generated within the sample groups. The Euclidean Mean Distance (EMD) between predictive values represents a measure of dissimilarity. (A) The mean EMD between intermediate prediction values for data from breath analysis of fourteen persons with cirrhosis is shown. (B) The average and standard deviation of EMD in a set of biological replicates obtained over multiple days, technical replicates from a healthy individual, and samples from persons with or without cirrhosis.

A

Pt	A	B	C	D	E	F	G	H	I	J	K	L	M	N
A	0.0													
B	1.5	0.0												
C	1.6	1.8	0.0											
D	2.7	2.9	3.1	0.0										
E	1.6	1.9	1.7	3.0	0.0									
F	1.5	1.7	1.9	2.0	1.9	0.0								
G	1.6	1.8	1.8	2.7	2.0	1.5	0.0							
H	1.6	1.8	1.9	2.8	2.1	1.8	1.7	0.0						
I	2.2	2.3	2.5	1.3	2.5	1.6	2.2	2.3	0.0					
J	2.5	2.8	2.9	1.2	2.7	1.9	2.6	2.7	1.4	0.0				
K	1.6	1.7	1.8	2.5	2.0	1.4	1.2	1.6	2.0	2.4	0.0			
L	1.6	1.9	1.6	2.7	2.0	1.7	1.9	1.4	2.2	2.6	1.8	0.0		
M	1.4	1.5	1.8	2.8	1.9	1.7	1.6	1.7	2.1	2.7	1.7	1.8	0.0	
N	1.5	2.0	1.4	2.7	1.5	1.6	1.9	2.0	2.3	2.6	1.9	1.8	2.0	0.0

B

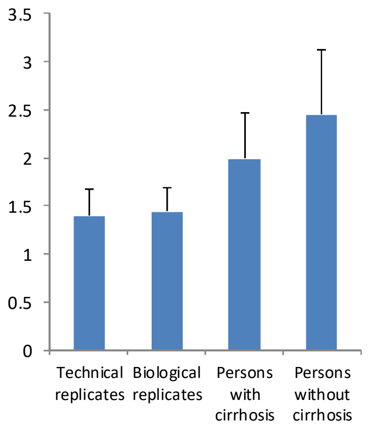

Supplement: S4 Fig — (PDF) [file pone.0260098.s004.pdf]
