# Supplementary material for: Machine learning analysis of volatolomic profiles in breath can identify non-invasive biomarkers of liver disease: A pilot study
Source: PLoS One. 2021 Nov 30;16(11):e0260098. doi: 10.1371/journal.pone.0260098 (PMC8631657; doi:10.1371/journal.pone.0260098)
Supplement: S5 Fig — (PDF) [file pone.0260098.s005.pdf]

Supplementary Figure 5: Performance of classifier models in distinguishing across disease stages.

Models SC-1A based on Subspace KNN and SC-2A based on RUSBoosted Trees were trained and tested on optimal samples from patients with stage 0, 1, 2 or 3 disease and cirrhosis, but not including non-cirrhotic portal hypertension. The table represents performance of these models on an independent validation cohort for distinguishing between different stages.

SC-1A	S0 vs S1/S2/S3	S0 vs S1/S2	S0 vs S1	S0 vs S2	S0 vs S3
Accuracy	0.880	0.883	0.825	0.833	0.742
Sensitivity	0.949	0.977	0.958	1.000	0.867
Specificity	0.625	0.625	0.625	0.625	0.625
Precision	0.903	0.878	0.793	0.769	0.684
F Measure	0.926	0.925	0.868	0.870	0.765
AUROC	0.787	0.801	0.793	0.813	0.746

SC-2A	S0 vs S1/S2/S3	S0 vs S1/S2	S0 vs S1	S0 vs S2	S0 vs S3
Accuracy	0.853	0.839	0.756	0.892	0.828
Sensitivity	0.881	0.870	0.760	1.000	0.923
Specificity	0.750	0.750	0.750	0.750	0.750
Precision	0.929	0.909	0.826	0.840	0.750
F Measure	0.904	0.889	0.792	0.913	0.828
AUROC	0.816	0.810	0.755	0.875	0.837
